# Early administration of anamorelin improves cancer cachexia in gastrointestinal cancer patients: an observational study

**DOI:** 10.1038/s41598-024-81195-3

**Published:** 2024-12-03

**Authors:** Toshihiko Matsumoto, Sien Cho, Akio Nakasya, Hiroki Nagai, Hironaga Satake, Hisateru Yasui

**Affiliations:** 1 Department of Medical Oncology, Ichinoimiya Nishi Hospital, 1, Kaimei Aza Hira, Ichinomiya City, Aichi 4940001 Japan; 2 Department of Medical Oncology, Kobe City Medical Center General Hospital, Kobe City, Hyogo Japan; 3 Department of Medical Oncology, Kochi Medical School, Kohasu, Oko-cho, Nankoku-City, Kochi Japan

**Keywords:** Anamorelin, Retrospective study, Cachexia, Cancer, Cancer, Gastroenterology

## Abstract

To report the efficacy of anamorelin in patients with colorectal and gastric cancer with cachexia and in those receiving systemic chemotherapy. We retrospectively collected real-world data from patients diagnosed with colorectal and gastric cancers experiencing cachexia who were treated with anamorelin. We evaluated the efficacy of treatment by measuring the improvements in appetite and body weight (BW) gain. Between June 2021 and October 2022, 43 cancer patients with cachexia—23 with gastric cancer and 20 with colorectal cancer—were treated with anamorelin. Median observation period was 7.3 months. The participants were 25 males with median age of 71 years and median BMI of 19.7. The ECOG PS distribution was 4, 33, 6 for grades 0, 1, and 2, respectively. Seven patients received supportive care only, while 36 received anamorelin with chemotherapy. Thirty-four had received chemotherapy previously (≤ 2 regimens) and nine had received ≥ 3 regimens. Median anamorelin treatment duration was 2.8 months; overall survival was 7.3 months. After 3 weeks, 24 experienced appetite improvement and 21 gained weight; after 12 weeks, 20 experienced appetite improvement and 15 gained weight. Multivariate analysis showed that anamorelin treatment before second-line chemotherapy and colorectal cancer correlated with appetite improvement and weight gain at 3 weeks. In the univariate analysis, anamorelin treatment before second-line chemotherapy correlated with weight gain at 12 weeks and with improved overall survival in patients with weight gain at 12 weeks. Early anamorelin treatment contributes to appetite improvement and BW gain in colorectal and gastric cancers with cachexia.

## Introduction

Patients with advanced cancer may experience a systemic condition known as cancer cachexia, which is characterized by weight loss caused by changes in fat and skeletal muscle tissue mass that can weaken the ability of the patient to withstand anticancer treatment, diminish the quality of life, and worsen the prognosis ^1,2^. Cachexia affects approximately 80% of patients with advanced cancer ^3,4^ and accounts for approximately 30% of cancer-related deaths ^5–9^. Furthermore, up to 80% of patients with advanced gastric cancer (GC) develop cachexia ^10^, as do 37–50.4% of patients with unresectable colorectal cancer (CRC) ^11,12^.

Cancer cachexia causes metabolic changes that negatively affect the quality of life of patients and reduce their tolerance to chemotherapy ^13,14^. Furthermore, cancer cachexia is a poor prognostic factor in patients with cancer, including those with GC and CRC ^4,15,16^. The prevention and treatment of cachexia in patients with advanced cancer have become an important issue.

​Anamorelin is an oral ghrelin mimetic and selective agonist that exerts its action at the ghrelin receptor, improves appetite, and affects metabolism. Clinical trials in Japan have shown that anamorelin effectively manages cancer cachexia by increasing lean body mass, body weight (BW), and alleviating anorexia-related symptoms ^17–19^. Following these trials, anamorelin was approved in Japan in January 2021 for the management of cachexia in patients with non-small cell lung cancer (NSCLC), GC, CRC, and pancreatic cancer. However, the number of patients enrolled in clinical trials for gastrointestinal cancer was small, with only 45 cases of GC and CRC. Thus, the factors associated with the efficacy of anamorelin in treating cachexia in gastrointestinal cancers remain unclear.

Therefore, we conducted an observational study of patients with GC and CRC with cachexia who were treated with anamorelin to explore the effectiveness of anamorelin and the factors associated with its efficacy in real-world clinical practice.

## Materials and methods

### Patients

The study included patients with gastric and colorectal cancers experiencing cachexia who received anamorelin treatment at Kobe City Medical Center General Hospital in Hyogo, Japan, between June 2021 and October 2022. Cachexia was defined as a weight loss exceeding 5% within six months, along with at least two of the following symptoms: (i) fatigue or malaise; (ii) muscle weakness; and (iii) presence of at least one of the following conditions: C-reactive protein (CRP) levels > 0.5 mg/dl, hemoglobin (Hb) levels < 12 g/dl, and albumin (Alb) levels < 3.2 g/dl.

All data were retrospectively collected from the electronic medical records. All procedures were performed in accordance with institutional and national standards on human experimentation, as confirmed by the ethics committee of Kobe City Medical Center General Hospital, and with the Declaration of Helsinki of 1964 and later versions. The study protocol was approved by the Institutional Review Board of Kobe City Medical Center General Hospital (approval no.: zn230610). Informed consent was obtained from all patients as opt-out forms on the hospital website.

### Treatment

Anamorelin (100 mg; ADLUMIZ; Ono Pharmaceutical Co., Ltd.) was administered orally once daily.

### Evaluation and statistical analysis

Efficacy was assessed based on the improvements in appetite loss and BW gain after 3 and 12 weeks. BW gain is defined as an increase in body weight of 0.5 kg or more, and improvement in appetite is defined as cases where the score for question 8 'Did you have a good appetite?' from the Quality of Life Questionnaire for Patients with Cancer who Were Treated with Anticancer Drugs (QoL-ACD) improved from baseline. Toxicity was assessed using the Common Terminology Criteria for Adverse Events (CTCAE), version 4.1. Overall survival (OS) was measured from the start of anamorelin treatment until death, with patients alive or with missing data at the cut-off point being censored. Time-to-treatment failure (TTF) was calculated from anamorelin initiation to confirmed discontinuation, excluding patients without discontinuation information. OS and TTF were estimated via the Kaplan–Meier method. Differences between patients with GC and CRC were analyzed using Fisher’s exact test. Logistic regression analysis was used to determine the odds ratios for appetite improvement and BW gain. Statistical analyses were conducted using JMP version 12 (SAS Institute Inc., Cary, NC, USA).

## Results

Between June 2021 and October 2022, 43 patients received anamorelin. The median observation time was 7.3 months (range: 0.6–29.2); patient characteristics are presented in Table 1. Twenty-three patients (53%) had GC and 20 (47%) had CRC. Twenty-five patients (58%) were male, the median age was 71 years (range: 47–85), the median body mass index (BMI) was 19.7 (range: 11.8–28.5), and the ECOG PS 0/1/2 ratio was 4/33/6 patients. Thirty-six patients (82%) received systemic chemotherapy before anamorelin administration. Six patients (14%) were administered anamorelin before their first-line treatment, while 18 (42%) received it during their first-line treatment. Ten patients (23%) received it during their second-line treatment and nine (21%) during a third or subsequent line of treatment.

**Table 1:** Patients Background.

		All (n = 43)	Gastric (n = 23)	Colorectal (n = 20)	p
Age	Median(range)	71 (47–85)	73 (47–85)	71 (50–85)	0.8933
Sex	Male	25 (58%)	13 (57%)	12 (60%)	0.8176
ECOG PS	0	4 (9%)	3 (13%)	1 (5%)	0.6481
	1	33 (77%)	17 (74%)	16 (80%)	
	2	6 (14%)	3 (13%)	3 (15%)	
Primary site	Gastric	23 (53%)			
	Colorectal	20 (47%)			
Peritoneal dissemination	Yes	22 (51%)	17 (74%)	5 (25%)	0.0022
Ascites	Yes	23 (53%)	18 (78%)	5 (25%)	0.0007
Primary site resection	Yes	18 (42%)	6 (26%)	12 (60%)	0.0246
Body mass index	Median (range)	19.7 (11.8–28.5)	18.7 (11.8–26.5)	20.4 (17.8–28.5)	0.0149
Serum Hb (g/dl)	Median (range)	10.3 (5.6–14.5)	10.4 (7.1–14.5)	10.0 (5.6–13.1)	0.3938
Serum Albumin (g/dl)	Median (range)	2.9 (1.6–4)	2.8 (1.6–4)	2.9 (1.7–3.6)	0.9415
Neutrophil lymphocyte ratio(NLR)	Median (range)	2.8 (0.2–14.3)	2.5 (0.3–7.9)	4.2 (0.3–14.3)	0.0139
Serum CRP (mg/dl)	Median (range)	0.95 (0.02–17.1)	0.45 (0.05–8.37)	3.53 (0.02–17.1)	0.0018
Modified glasgow prognostic score(mGPS)	0	8 (19%)	4 (17%)	4 (20%)	0.0005
	1	17 (40%)	15 (65%)	2 (10%)	
	2	18 (42%)	4 (17%)	14 (70%)	
Number of metastatic organ	2 or more	26 (60%)	11 (48%)	15 (75%)	0.0691
Number of prior chemotherapy regimen	0	6 (14%)	5 (22%)	1 (5%)	0.5333
	1	18 (42%)	10 (43%)	8 (40%)	
	2	10 (23%)	5 (22%)	5 (25%)	
	3 or more	9 (21%)	3 (13%)	6 (30%)	
Systemic therapy for cancer	Chemotherapy	36 (82%)	18 (78%)	18 (90%)	0.2983
	Best supportive care	7 (18%)	5 (22%)	2 (10%)	

*ECOG* Eastern Cooperative Oncology Group, *PS* performance status.

When comparing the backgrounds of patients with GC with those of patients with CRC, several distinctions emerged. Patients with GC exhibited low BMI (18.7 vs. 20.4, p = 0.0149), high prevalence of peritoneal dissemination (74% vs. 25%, p = 0.0022), and ascites compared to those with CRC (78% vs. 25%, p = 0.0007). Conversely, patients with CRC had a high rate of undergoing surgery (60% vs. 26%, p = 0.0246) and presented with elevated neutrophil-to-lymphocyte ratio (NLR) (4.2 vs. 2.5, p = 0.0139) and modified Glasgow Prognostic Score (mGPS: p = 0.0005) when compared to those with GC.

### Efficacy

**Fig. 1:**
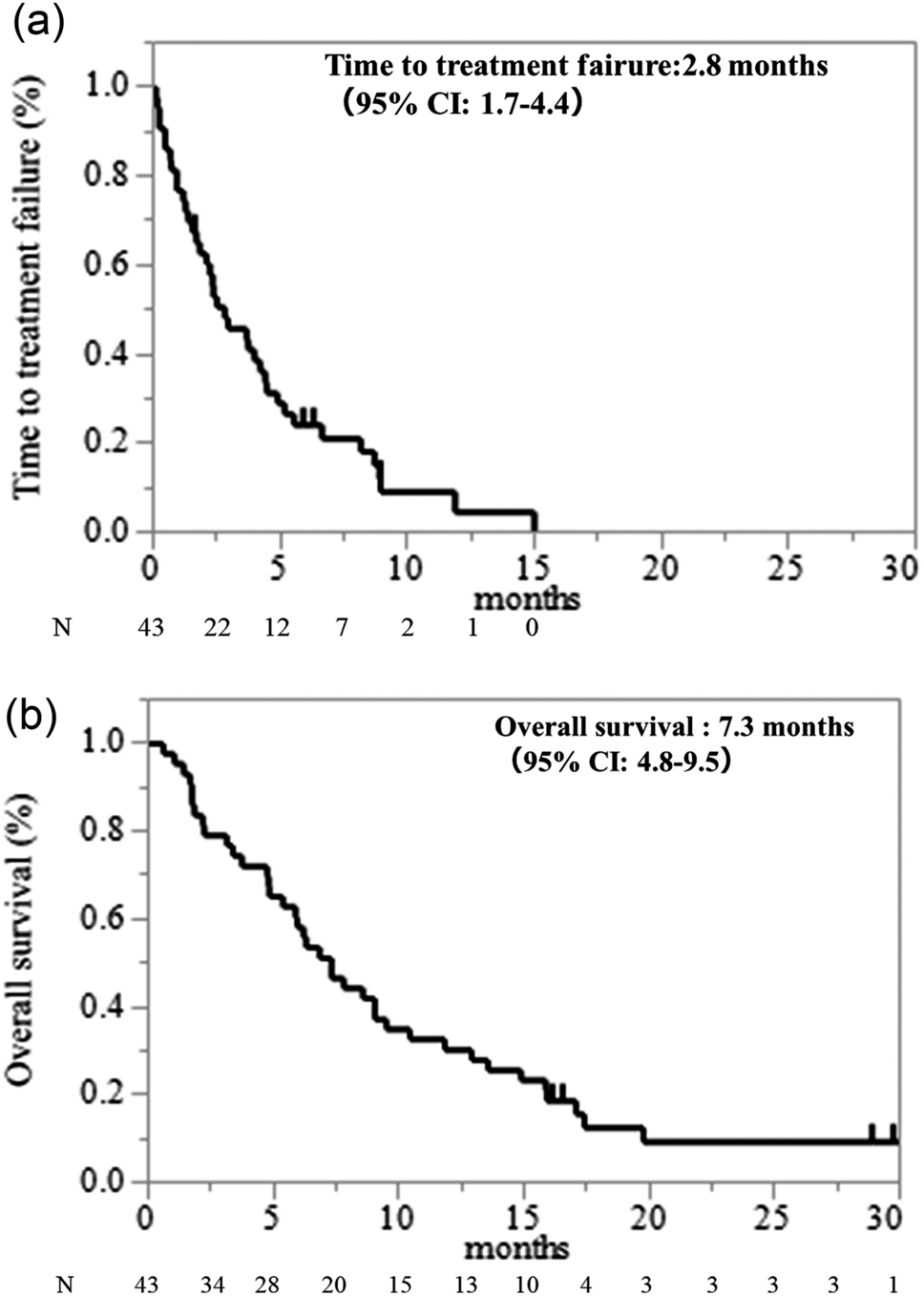
Kaplan–Meier survival curves for (**a**) time-to-treatment failure and (**b**) overall survival.

**Fig. 2:**
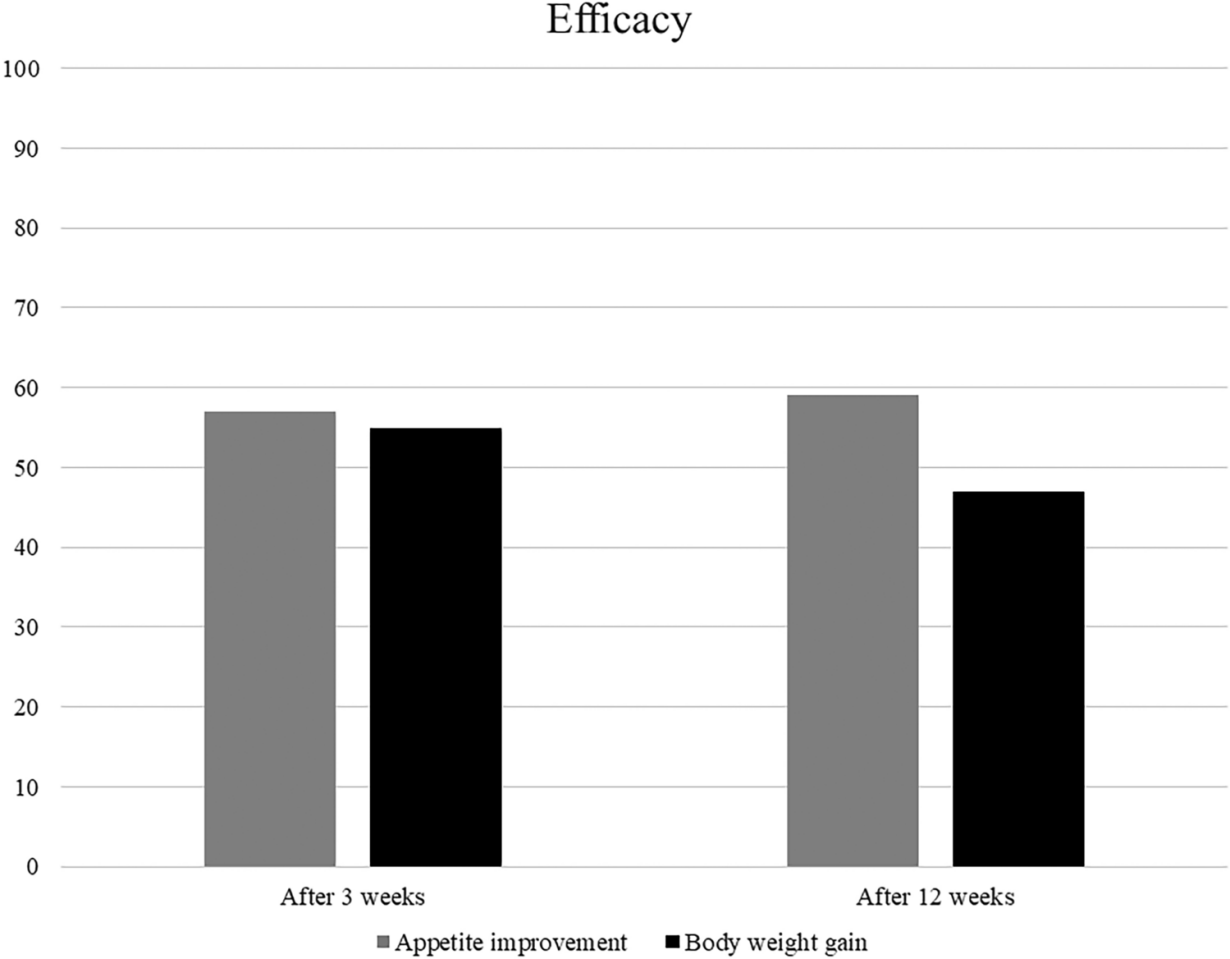
Efficacy of anamorelin after 3 and 12 weeks.

**Fig. 3:**
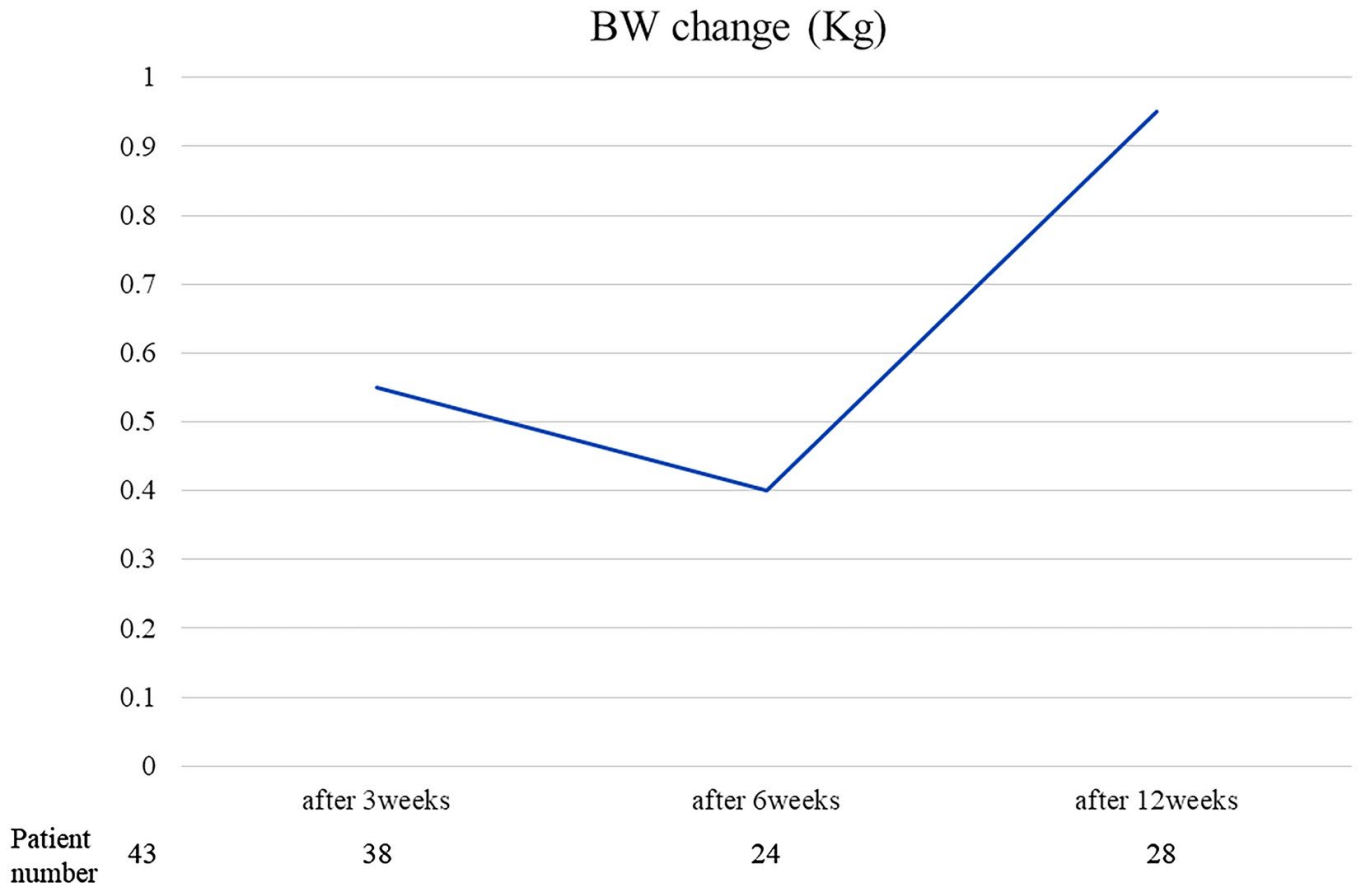
Body weight change after 3, 6 and 12 weeks.

**Fig.4:**
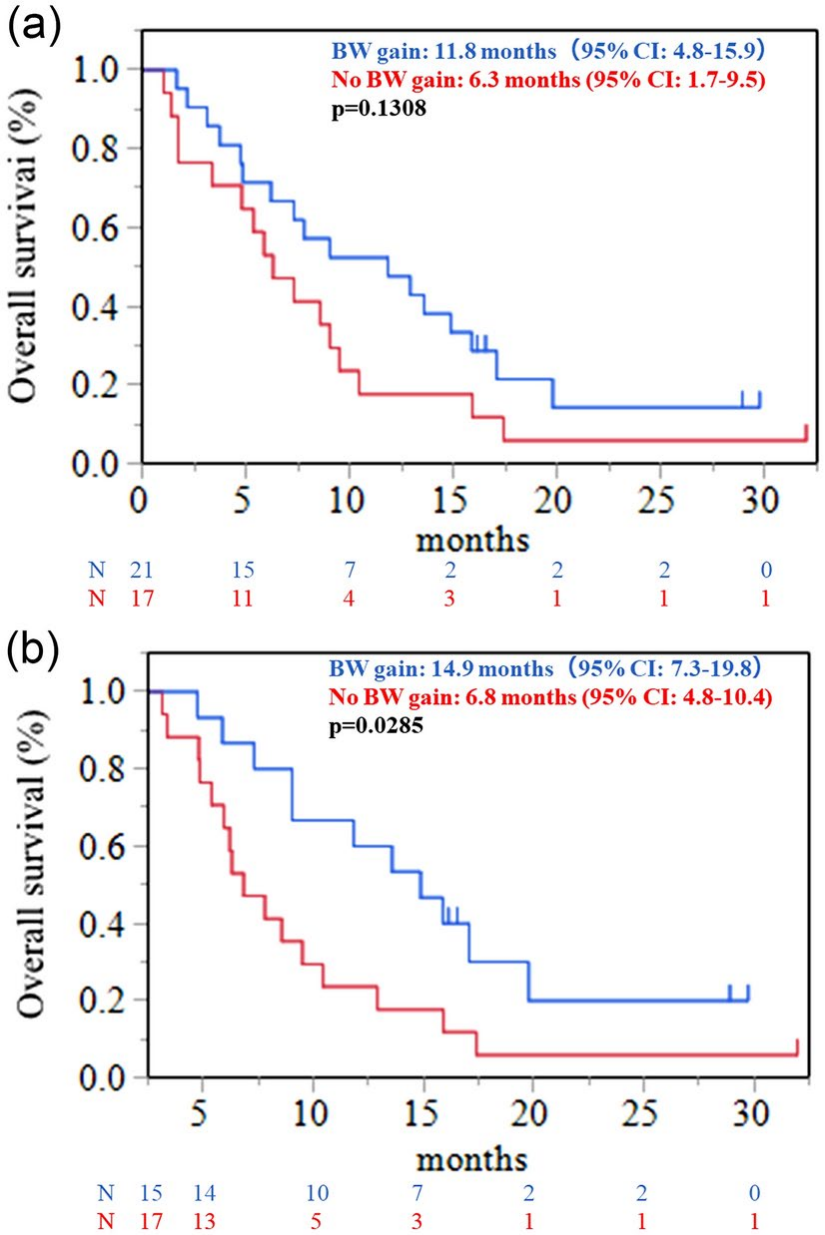
Kaplan–Meier survival curves of overall survival (**a**) with or without body weight gain after 3 and (**b**) 12 weeks.

Median time to TTF for anamorelin was 2.8 months (95% confidence interval (CI): 1.7–4.4), the median OS was 7.3 months (95% CI 4.8–9.5), and nine patients (21%) had died at 12 weeks (Fig. 1). After 3 weeks, 42 patients received anamorelin, among whom 24 (57%) experienced appetite improvement and 21 (55%) experienced BW gain. Notably, the median BW change after 3 weeks was 0.55 kg (range: -6 to 8.5). After 12 weeks, among the 34 patients who received anamorelin, 20 (59%) experienced appetite improvement and 15 (47%) experienced BW gain (Fig. 2). The median BW changes at 3 and 12 weeks were 0.55 kg (range: -6 to 8.5) and 0.95 kg (range: -6 to 9.7), respectively. The median BW gain was 0.55 kg at 3 weeks, 0.4 kg at 6 weeks, and 0.95 kg at 12 weeks (Fig. 3 and supplement Fig. 1).An analysis of the relationship between BW gain and OS (Fig. 4) revealed a positive trend in OS when BW gain was observed at 3 weeks (11.8 vs. 6.3 months, p = 0.1308), and OS significantly improved when BW gain was observed at 12 weeks (14.9 vs. 6.8 months, p = 0.0285).

### Univariate and multivariate analyses of anamorelin efficacy

We analyzed the factors that correlated with appetite improvement and BW gain after 3 and 12 weeks (Tables 2 and 3). In the univariate analysis, anamorelin administration during second-line chemotherapy or before significantly correlated with appetite improvement (Odds Ratio (OR): 7.0 [95% CI 1.42–52.59], p = 0.02) and BW gain at 3 weeks (OR: 8.33 [95% CI 1.16–169.98], p = 0.0337). In patients with CRC, there was a significant increase in BW gain after 3 weeks (OR: 4.33 [95% CI 1.11–19.75], p = 0.034). In the multivariate analysis, anamorelin administration during second-line chemotherapy or sooner (OR: 14.27 [95% CI 1.82–193.01], p = 0.0098), CRC (OR: 8.70 [95% CI 1.41–86.77], p = 0.0181), and BW gain were identified as important factors, and GPS 0 or 1 (OR: 15.38 [95% CI 1.68–512.54], p = 0.0123) significantly correlated with appetite improvement after 3 weeks. Furthermore, anamorelin administration during the second-line chemotherapy or before (OR: 37.87 [95% CI 3.77–1317.22], p = 0.0007), CRC (OR: 23.65 [95% CI 1.49–1511.23], p = 0.0224), and GPS 0/1 (OR: 15.38 [95% CI 1.68–512.54], p = 0.0123) significantly correlated with BW gain after 3 weeks. At 12 weeks, in the univariate analysis, only anamorelin administration at second-line chemotherapy or before significantly correlated with BW gain (OR: 6.59 [95% CI 1.02–129.86], p = 0.05). In the multivariate analysis at 12 weeks, none of the factors showed a significant correlation with BW gain.None of the factors showed a significant correlation with appetite improvement at 12 weeks in univariate and multivariate analysis.

**Table 2:** Univariate and multivariate analysis of appeteite improvement.

	After 3 weeks		After 12 weeks	
	Univariate analysis	Multivariate analysis	Univariate analysis	Multivariate analysis
Gastric vs colorectal	2.36 (95% CI 0.68–8.82)	8.70 (95% CI 1.41–86.77)	1.33 (95% CI 0.34–5.44)	2.35 (95% CI 0.44–15.86)
	p = 0.1768	p = 0.0181	p = 0.6810	p = 0.3237
3rd or later vs 2nd or faster	7.0 (95% CI 1.42–52.59)	14.27 (95% CI 1.82–193.01)	3.6 (95% CI 0.59–29.47)	5.02 (95% CI 0.69–51.21)
	p = 0.02	p = 0.0098	p = 0.1644	p = 0.11
ECOG PS 2 vs 0/1	0.625 (95% CI 0.08–3.64)	0.85 (95% CI 0.08–7.33)	3.17 (95% CI 0.27–72.58)	4.60 (95% CI 0.29–132.89)
	p = 0.6068	p = 0.8831	p = 0.3510	p = 0.2766
mGPS 2 vs 0/1	0.75 (95% CI 0.21–2.59)	1.40 (95% CI 0.27–7.79)	1.13 (95% CI 0.28–4.55)	1.15 (95% CI 0.19–6.99)
	p = 0.6552	p = 0.6911	p = 0.8678	p = 0.8779
NLR ≧median vs > median	2.80 (95% CI 0.81–10.54)	3.21 (95% CI 0.71–17.01)	1.86 (95% CI 0.46–7.74)	1.73 (95% CI 0.38–8.24)
	p = 0.1060	p = 0.1318	p = 0.3822	p = 0.4756

*ECOG* Eastern Cooperative Oncology Group, *PS* performance status, *mGPS* modified glasgow prognostic score, *NLR* neutrophil lymphocyte ratio.

**Table 3:** Univariate and multivariate analysis of body weight gain.

	After 3 weeks		After 12 weeks	
	Univariate analysis	Multivariate analysis	Univariate analysis	Multivariate analysis
Gastric vs colorectal	4.33 (95% CI 1.11–19.75)	37.87 (95% CI 3.77–1317.22)	0.85 (95% CI 0.22–3.10)	2.17 (95% CI 0.36–18.43)
	p = 0.034	p = 0.0007	p = 0.8041	p = 0.4089
3rd or later vs 2nd or faster	8.33 (95% CI 1.16–169.98)	23.65 (95% CI 1.49–1511.23)	6.59 (95% CI 1.02–129.86)	8.81 (95% CI 0.94–218.70)
	p = 0.0337	p = 0.0224	p = 0.05	p = 0.0564
PS 2 vs 0/1	0.27 (95% CI 0.01–2.04)	0.14 (95% CI 0.003–2.44)	1.22 (95% CI 0.11–27.60)	2.7922e-8 (95% CI 0–2.83)
	p = 0.2152	p = 0.1951	p = 0.8759	p = 0.1244
GPS 2 vs 0/1	1.44 (95% CI 0.39–5.40)	15.38 (95% CI 1.68–512.54)	2.54 (95% CI 0.66–11.19)	5.70 (95% CI 0.91–57.02)
	p = 0.578	p = 0.0123	p = 0.1766	p = 0.0641
NLR ≧median vs > median	1.57 (95% CI 0.43–5.89)	1.71 (95% CI 0.28–11.50)	1.02 (95% CI 0.25–4.15)	0.77 (95% CI 0.13–4.11)
	p = 0.4909	p = 0.5562	p = 0.9823	p = 0.7643

*ECOG* Eastern Cooperative Oncology Group, *PS* performance status, *mGPS* modified glasgow prognostic score, *NLR* neutrophil lymphocyte ratio.

### Safety

Adverse events (grade 1) were observed in five patients—nausea in two patients, dizziness in two, and edema in one. Forty-two patients (98%) discontinued anamorelin, the reasons for which are listed in Table 4. Twenty-one patients (50%) discontinued treatment because of progressive disease, and only one patient (2%) discontinued treatment because of an adverse event—dizziness. Eleven patients discontinued treatment due to effective response of anamorelin.. The rate of discontinuation due to the lack of therapeutic effect was significantly lower in patients who received anamorelin with second-line therapy or prior (OR: 5.7497e−9 [95% CI not evaluated–0], p = 0.0135) than in those who received anamorelin with third or later line chemotherapy.

**Table 4:** Reason of discontinuation.

Cancer progression	21 (48.8%)
Poor response	9 (20.9%)
Adverse events	1 (2.3%)
Effective response	11 (25.6%)
Lost of follow up	1 (2.3%)

## Discussion

To the best of our knowledge, this is the first study to investigate the relationship between patient background and anamorelin efficacy in GC and CRC. In a clinical trial of cachexia in GC, the rate of increase in lean body mass after 12 weeks was 63.3% ^17^. During the overall evaluation of cancer cachexia, including GC/CRC/pancreatic cancer and NSCLC, BW gain and appetite improvement were observed at rates of 28.6% and 62.5% ^20^, respectively, after 3 weeks, and 40.4% and 57.3%, respectively, after 12 weeks. In the present study, BW gain and appetite improvement were observed at rates of 55% and 57% at 3 weeks and at rates of 47% and 59% at 12 weeks, respectively. These results suggest that anamorelin may be beneficial in the clinical setting for treating cachexia in patients with GC and CRC.

There are no specific discontinuation criteria for anamorelin in cancer cachexia. In post-marketing surveillance conducted in Japan, it was reported that 30.8% discontinued due to cancer progression, 22.1% due to poor response, 15.9% due to adverse events, and 9.6% due to effective response^21^. In our study, 48.8% discontinued due to cancer progression, 20.9% due to poor response, 25.6% due to effective response and 11.6% discontinued due to adverse events. In our study, as well as in the preceding post-marketing surveillance, no specific criteria for discontinuing anamorelin were established. Given that there were no apparent differences in the reasons for discontinuation between the two studies, it can be inferred that the timing of anamorelin discontinuation in our study was not atypical compared to existing research.

In the present study, the efficacy of anamorelin after 3 weeks was lower in patients with GC than in those with CRC. Despite worse background factors, such as mGPS and NLR, in patients with CRC than in those with GC, the multivariate analysis identified unfavorable factors for BW gain and appetite improvement at 3 weeks. A plausible explanation for this discrepancy could be the greater occurrence of peritoneal dissemination and ascites in patients with GC, which may lead to a potentially reduced impact of anamorelin. However, as no significant difference was observed between patients with GC and CRC after 12 weeks, anamorelin may have a consistent effect on cachexia in both types of cancer.

The administration of anamorelin as a second-line or earlier treatment resulted in significant appetite improvement and BW gain at 3 weeks, with a favorable trend also observed in appetite improvement and BW gain at 12 weeks. One possible reason could be differences in the therapeutic efficacy of chemotherapy. In this study, the response rate was 35.3% and disease control rate was 64.7% up to second-line chemotherapy, whereas in third-line chemotherapy and beyond, the response rate was 0% and disease control rate was 25%. These results suggest that achieving the effects of anamorelin for cancer cachexia may also depend significantly on concurrent treatments that achieve cancer control.

Cachexia has been suggested to progress from "pre-cachexia" to “cachexia” and then advance to "refractory cachexia," becoming irreversible ^7,22^. Our study suggests that in situations where treatment is extended over a long period, several patients experience cachexia progression, which makes anamorelin less efficacious. Therefore, it is desirable to initiate anamorelin treatment at the earliest stage of cachexia in GC and CRC. Cachexia in the pre-cachexia or early cachexia stage shows potential for improvement with treatment. However, once cachexia advances to refractory stages, it may become irreversible despite treatment.

Fearlon et al. define “refractory cachexia” as having poor responsiveness to treatment, active catabolic processes, a low performance status (WHO score 3 or 4), and a life expectancy of less than 3 months ^1^. However, there are still no clear diagnostic criteria for refractory cachexia. In this study, we investigated the efficacy of anamorelin administered at the timing before second-line treatment and after third-line treatment. As treatment progresses in advanced cancer, therapeutic effects diminish and prognosis worsens, suggesting an increasing prevalence of refractory cachexia with advancing treatment stages. Early administration of anamorelin after chemotherapy initiation is considered crucial in patients with advanced GC or CRC complicated by cachexia, and the results of this study are thought to support this approach.

Cancer cachexia refers to systemic inflammation caused by advanced cancers. In recent years, mGPS and NLR have been used as indicators of systemic inflammation in GC and CRC ^23–28^. In this study, we explored the relationship between these two factors and the effects of anamorelin. In patients with mGPS 2, the multivariate analysis showed a negative correlation with BW gain at 3 weeks and a trend toward low BW gain at 12 weeks. Silva et al. conducted an observational study that targeted patients with advanced cancer receiving palliative care, wherein they defined cachexia using mGPS. They classified patients with an mGPS of 2 as having refractory cachexia and reported that such patients had low BW, low BMI, poor patient-generated subjective global assessment short-form scores, and poor prognosis ^29^. These results suggest that mGPS is crucial for understanding the state of cancer cachexia; thus, initiating interventions for cancer cachexia before it progresses to mGPS of 2 is recommended.

The effect of anamorelin on treatment outcomes, such as OS, in patients with advanced cancer with cachexia is not yet understood. In the present study, we observed a correlation between favorable OS and BW gain at 12 weeks in patients with cachexia, GC, and CRC who received anamorelin. This result suggests the possibility of improved prognosis in cancer patients with cachexia in whom BW gain has been observed because of anamorelin. However, despite receiving anamorelin treatment, BW gain after 12 weeks remained at 47%, underscoring the importance of considering further treatment strategies for cancer cachexia. Sugiyama et al. conducted a study on primary chemotherapy and nutritional support for unresectable advanced GC and found that nutritional support contributed to an extension in TTF, according to their multivariate analysis ^30^. Additionally, their study identified low BMI and an mGPS of 1 or higher as poor prognostic factors. Therefore, the addition of other treatments, such as nutritional support, is important for patients with GC and CRC and cachexia.

Our study has several limitations. First, this was an observational study with a small number of patients and interpretation of the results from univariate and multivariate analyses of factors associated with efficacy requires careful consideration. Second, owing to the single-arm design, there was a lack of comparative groups. Additionally, there was variability in the treatment lines among the study population, which made it difficult to analyze OS. Thus, further research is necessary to explore the effects of anamorelin on cachexia associated with GC and CRC as well as to develop better treatments for improving cachexia.

In conclusion, anamorelin showed similar efficacy in patients with GC and CRC with cachexia in a clinical setting. The early administration of anamorelin before or during second-line therapy was found to contribute to appetite improvement and BW gain in patients with cachexia with GC and CRC.

## Supplementary Information

Supplementary Legends.

Supplementary Figure 1.

## Data Availability

The datasets used and/or analyzed during the current study available from the corresponding author on reasonable request.
