# Supplementary material for: Early administration of anamorelin improves cancer cachexia in gastrointestinal cancer patients: an observational study
Source: Sci Rep. 2024 Dec 3;14:30017. doi: 10.1038/s41598-024-81195-3 (PMC11612480; doi:10.1038/s41598-024-81195-3)
Supplement: Supplementary file 1 — Supplementary Legends. [file 41598_2024_81195_MOESM1_ESM.docx]

supplementary information

supplementary figure.1

Body weight change after 3, 6 and 12 weeks of treatment with anamorelin

1. Primary site
2. Treatment line when administered with anamorelin
3. ECOG PS
4. GPS
5. NLR
