# Supplementary figures and images for: Early administration of anamorelin improves cancer cachexia in gastrointestinal cancer patients: an observational study
Source: Sci Rep. 2024 Dec 3;14:30017. doi: 10.1038/s41598-024-81195-3 (PMC11612480; doi:10.1038/s41598-024-81195-3)

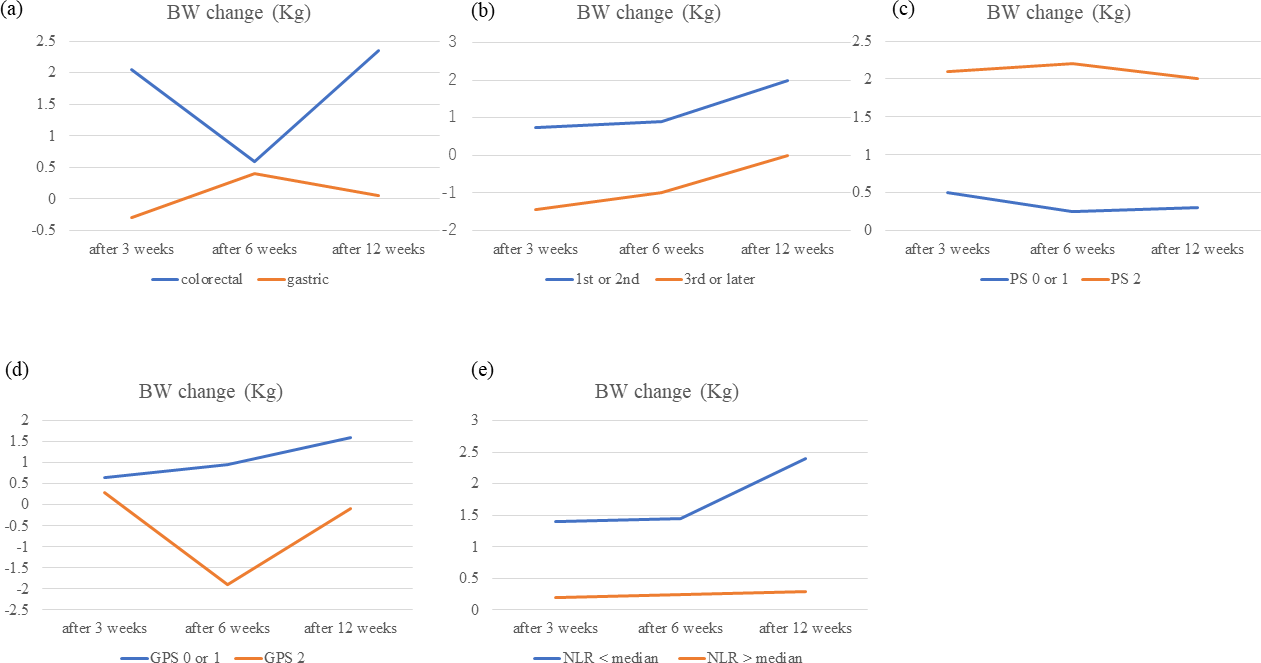

Supplement: Supplementary file 2 — Supplementary Figure 1. [file 41598_2024_81195_MOESM2_ESM.tif]
